# A clinical study of pegylated recombinant human granulocyte colony stimulating factor (PEG-rhG-CSF) in preventing neutropenia during concurrent chemoradiotherapy of cervical cancer

**DOI:** 10.1186/s12885-021-08364-9

**Published:** 2021-06-02

**Authors:** Dongling Zou, Mingfang Guo, Qi Zhou

**Affiliations:** Chongqing University Cancer Hospital, Shapingba district, Chongqing, 400030 China

**Keywords:** Cervical Cancer, Concurrent Radiochemotherapy, Neutropenia, Pegylated recombinant human granulocyte colony stimulating factor

## Abstract

**Purpose:**

To evaluate the effectiveness and safety of pegylated recombinant human granulocyte colony stimulating factor (PEG-rhG-CSF) in preventing neutropenia during chemoradiotherapy in patients with cervical cancer.

**Methods:**

From August 2018 to April 2020, 60 patients who were pathologically confirmed as cervical cancer were randomly divided into two groups at a ratio of 2:1: PEG-modified-rhG-CSF experimental group and control group. The primary endpoints were the incidence of grade 3–4 neutropenia. Secondary endpoints included the duration of grade 3–4 neutropenia, the incidence of grade 4 neutropenia, the incidence of febrile neutropenia (FN), delay rate of chemotherapy, prolonged time of chemotherapy, time to complete radiotherapy and safety.

**Results:**

The incidence of grade 3–4 neutropenia in the experimental group was significantly lower than the control group (10% vs. 77.78%, *P* < 0.001). However, there was no statistical significance between the two groups in the duration of grade 3–4 neutropenia (3.75 days vs. 5.07 days, *P* = 0.871). The experimental group was better than the control group in the incidence of grade 4 neutropenia, the incidence of FN and delay rate of chemotherapy, and the difference was statistically significant (*P* < 0.05). Besides, the prolonged time of chemotherapy and the time to complete radiotherapy in the experimental group were less than those in the control group, but the difference was not statistically significant (*P* > 0.05). The incidence of adverse events in the experimental group and control group were 55.00 and 94.44%, respectively, and the difference was statistically significant (*P* = 0.003).

**Conclusion:**

PEG-rhG-CSF preventive treatment used in the course of chemoradiotherapy for patients with cervical cancer can reduce the incidence of neutropenia and improve the incidence of delayed chemotherapy cycles.

**Trial registration:**

ClinicalTrials.gov, NCT04542356. Registered 9 September 2020 - Retrospectively registered.

## Introduction

Cervical cancer is the third most common cancer among women in the world, and the most common cancer among women in Eastern and Central Africa [1, 2]. According to reports, the incidence and mortality of cervical cancer are incredibly high, accounting for about 86% of the deaths from cervical cancer worldwide. According to the 2018 International Federation of Gynecology and Obstetrics (FIGO) staging recommendation, the preferred treatment for patients with stage IIB ~ IVA cervical cancer is concurrent radiotherapy and chemotherapy (chemoradiotherapy). Studies have shown that compared with the same dose of radiotherapy, the five-year overall survival rate of chemoradiotherapy has increased by 6% [3]. Chemoradiotherapy can improve the efficacy and survival rate of cervical cancer, but it increases the incidence of acute blood adverse reactions, such as acute blood toxicity and neutropenia [4].

Neutropenia caused by chemoradiotherapy is often treated with recombinant human granulocyte colony stimulating factor (rhG-CSF) in clinical practice [5–7]. The circulating half-life (t1/2) of rhG-CSF is about 3–6 h and requires daily administration [8–10]. Pegylated modified recombinant human granulocyte colony stimulating factor (PEG-modified-rhG-CSF) is the long-acting form of rhG-CSF with t1/2 of about 42–62 h, which is a covalent combination of rhG-CSF and polyethylene glycol (PEG) [9, 11]. PEG-rhG-CSF mainly acts on hematopoietic cells, including promonocytes, eosinophils, lymphocytes, erythrocytes, and megakaryocytes, by binding to G-CSF receptor on the cell surface, thereby stimulating cell proliferation, differentiation, and activation of terminal cell functions, without altering the aggregation and bind pattern compared with rhG-CSF [9]. A multicenter prospective study indicated that the use of PEG-rhG-CSF for primary prevention can significantly reduce the incidence of febrile neutropenia (FN) caused by chemotherapy [12]. Although PEG-rhG-CSF for primary prevention can benefit patients, most of the evidence comes from chemotherapy, there are few clinical practice studies on concurrent chemoradiotherapy [13–15]. This current study firstly analyzed the efficacy and safety of PEG-rhG-CSF in the prevention of neutropenia caused by chemoradiotherapy in patients with cervical cancer. The results may provide a basis for the clinical application of PEG-rhG-CSF in the treatment process of chemoradiotherapy cycle in patients with cervical cancer.

## Patients and methods

From August 2018 to April 2020, a total of 60 patients with cervical squamous cell carcinoma diagnosed by histopathology and who were not initially treated with surgery (stage IIb-IIIb) were enrolled (clinical trials registration number NCT04542356). The study was approved by the Clinical Research Management Committee of Chongqing Cancer Hospital (No. 2019-linshen-027). The study received informed consent signed by all patients.

### Patients

The inclusion criteria were: women aged 18–70 years; pathologically confirmed and previously untreated with surgery Stage IIb-IIIb cervical cancer; Expected survival time ≥ 8 months; Eastern Cooperative Oncology Group (ECOG) performance score ≤ 1. The bone marrow hematopoietic function is normal, the absolute neutrophil count (ANC) ≥ 1.8 × 10^9^/L, platelet count (PLT) ≥ 100 × 10^9^/L, hemoglobin (Hb) ≥ 90 g/L and white blood cell count (WBC) ≥4.0 × 10^9^/L. All patients showed no obvious cardiac dysfunction through the ECOG examination. All patients must agree to take effective contraceptive measures during the study period and within 6 months after stopping treatment, and female patients of childbearing age must have a negative urine pregnancy test before treatment.

### Randomization and study treatment

According to the principle of 2:1 randomization, patients were divided into the experimental group and the control group. Patients in the experimental group were treated with PEG-rhG-CSF for primary prevention during concurrent chemoradiotherapy. Patients in the control group were treated with rhG-CSF when ANC < 1 × 10^9^/L occurred during concurrent chemoradiotherapy.

### Study procedures

#### Chemoradiotherapy regimen

The chemotherapy regimen was TP regimen: paclitaxel 150 mg/m^2^ on day 1 + cisplatin 35 mg/m^2^ on day 1–2. TP regimen was repeated every 3 weeks as a chemotherapy cycle, and radiotherapy was followed 24 h after the end of chemotherapy at each cycle. If patients experience intolerance with cisplatin, other platinums such as carboplatin can be used instead. All patients received two 3-week cycles of TP chemotherapy. Radiotherapy was given on the second day of chemotherapy at each cycle. The radiotherapy regimen is external beam radiotherapy (EBRT): 95% of planning tumor volume (PTV) received the prescribed dose of 45Gy/25 times, 5 times/week.

#### Dosing regimen

The experimental group was injected subcutaneously with 6 mg PEG-rhG-CSF (Qilu Pharmaceutical Co., Ltd.) 2 h after the end of radiotherapy at each cycle. The control group was not given PEG-rhG-CSF for treatment. If the patient has an ANC < 1 × 10^9^/L, 5μg/kg/d rhG-CSF (Qilu Pharmaceutical Co., Ltd.) was given for treatment subcutaneously, and both chemotherapy and radiotherapy were stopped until ANC ≥ 2 × 10^9^/L.

### Study outcomes

The primary outcome was the incidence of grade 3–4 neutropenia (defined as ANC < 0.5 × 10^9^/L). The secondary outcomes were the duration of grade 3–4 neutropenia (according to National Cancer Institute (NCI) common toxicity criteria, V4.03), the incidence of grade 4 neutropenia, the completion time of radiotherapy, delay rate of chemotherapy, prolonged time of chemotherapy, the incidence of FN and safety.

### Statistical analysis

Data analysis was performed using SAS9.4 software. For continuous data, if it obeys a normal distribution, the measured data were analyzed by t-test and the data were expressed as mean ± standard deviation. If it does not obey the normal distribution, analyze the measured data were analyzed by non-parametric rank sum test and the data were expressed as the median (Q1, Q3). For classified data, the measurement data were analyzed by *χ*^2^ test, and the data were expressed in frequency (percentage). *P* < 0.05 indicates that the difference was statistically significant.

## Results

### Patients

A total of 60 patients were included in this study between August 2018 and April 2020. Forty patients were randomly assigned to the experimental group and 20 patients were randomly assigned to the control group. However, two patients in the control group withdrew due to lack of treatment compliance. Table 1 summarizes the baseline characteristics of the patients. The average age of the experimental group and the control group were 54.25 years and 54.17 years, respectively, and the baseline absolute neutrophil count (ANC) was (4.26 ± 1.38) × 10^9^/L and (4.32 ± 1.74) × 10^9^/L (*P* = 0.889). According to FIGO staging, there were 19 patients with stage IIB and 21 patients with IIIA+IIIB in the experimental group. The control group included 3 patients with stage IIB and 15 patients with IIIA+IIIB. There was no significant difference between the two groups of patients in various baseline indicators. All patients received the second cycle of chemoradiotherapy without adjusting the dose, which was the same as the first cycle of chemoradiotherapy. In the second cycle of chemoradiotherapy, all patients in the control group used rhG-CSF for salvage treatment, while only 3 people in the experimental group used rhG-CSF and all of them received prophylactic PEG-rhG-CSF (Table 2).

**Table 1:** Baseline analysis

Characteristics	Experimental group (*N* = 40)	Control group (*N* = 18)	Statistics	*P*
Age	54.25 ± 7.48	54.17 ± 7.56	t = 0.04	0.969
Classification of diseases			–	–
CSEC	40 (100.00%)	18 (100.00%)		
FIGO staging			*χ*^2^ = 5.01	0.025
IIB	19 (47.50%)	3 (16.67%)		
IIIA+IIIB	21 (52.50%)	15 (83.33%)		
ECOG score			*χ*^2^ = 0.13	0.719
0	13 (32.50%)	5 (27.78%)		
1	27 (67.50%)	13 (72.22%)		
ANC baseline (×10^9^)	4.26 ± 1.38	4.32 ± 1.74	t = 0.14	0.889
Body surface area (m^2^)	1.54 ± 0.11	1.56 ± 0.17	t = 0.44	0.666
Second cycle precursor body surface area (m^2^)	1.54 ± 0.11	1.56 ± 0.17	t = 0.44	0.666

*CSEC* cervical squamous epithelium carcinoma, *ANC* Absolute neutophil count

**Table 2:** Exposure analysis

	Experimental group (*N* = 39)	Control group (*N* = 18)
First cycle		
Paclitaxel amount (mg)		
Median (Q1,Q3)	235.00 (220.00, 240.00)	225.00 (210.00, 240.00)
Mean ± SD	231.67 ± 16.69	227.94 ± 24.73
Paclitaxel dose (mg/m^2^)		
Median (Q1,Q3)	150.07 (149.34, 151.90)	149.33 (144.83, 150.00)
Mean ± SD	150.91 ± 7.26	146.78 ± 6.68
Cisplatin amount (mg)		
Median (Q1,Q3)	106.00 (100.00, 110.00)	100.00 (100.00, 110.00)
Mean ± SD	105.66 ± 9.70	102.89 ± 9.68
Cisplatin dose (mg/m^2^)		
Median (Q1,Q3)	69.18 (67.11, 70.51)	68.49 (66.12, 69.18)
Mean ± SD	68.73 ± 3.44	66.47 ± 5.14
Second cycle		
Whether to adjust the dose in the second cycle		
Yes	0	0
No	39 (100.00%)	18 (100.00%)
Whether to remedy the use of short-acting rhG-CSF		
Yes	3 (7.69%)	18 (100.00%)
No	36 (92.31%)	0

### Efficacy

#### The incidence and duration of grade 3–4 neutropenia

**Fig. 1:**
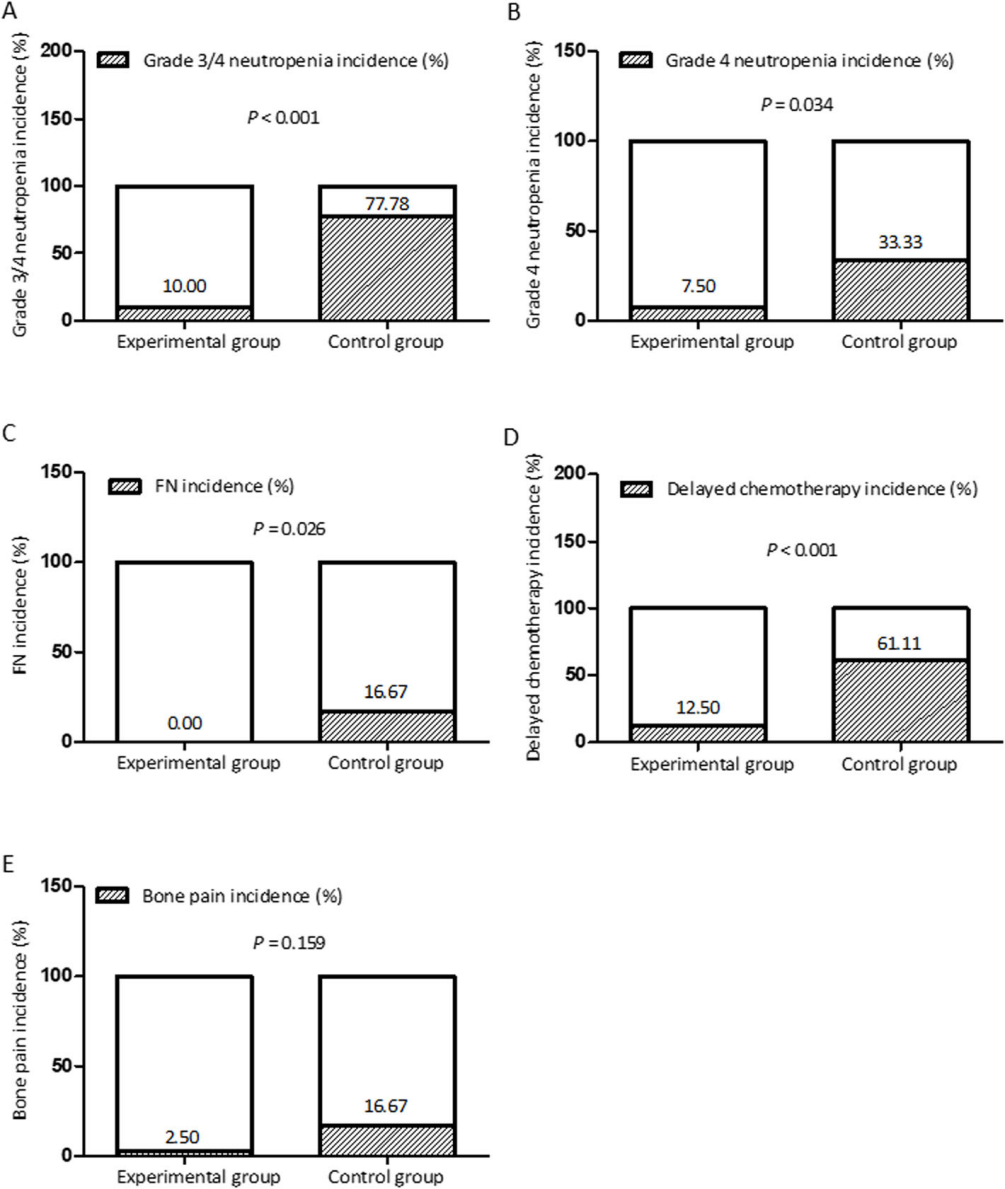
Summarized data presenting the analysis of endpoints

The primary endpoint, the incidence of grade 3–4 neutropenia, was significantly lower in experimental group (4/40; 10%) than that in the control group (14/18; 77.78%) (*P* < 0.001) (Table 3 and Fig. 1). The average duration of grade 3–4 neutropenia in the experimental group was 3.75 days and in the control group was 5.07 days. Although the duration of grade 3–4 neutropenia in the experimental group tended to decrease compared with the control group, the difference between the two groups was not statistically significant (*P* = 0.871) (Table 3).

**Table 3:** Analysis of endpoints

Endpoints	Experimental group (*N* = 40)	Control group (*N* = 18)	Statistics	*P*
Whether grade 3/4 neutropenia occurs			*χ*^2^ = 26.64	< 0.001
Yes	4 (10.00%)	14 (77.78%)		
No	36 (90.00%)	4 (22.22%)		
The duration of grade 3–4 neutropenia ^a^			Z = 0.16	0.871
n	4	14		
Median (Q1,Q3)	3.5 (3.0,4.5)	3.5 (3.0,9.0)		
Whether grade 4 neutropenia occurs			*χ*^2^ = 4.50	0.034
Yes	3 (7.50%)	6 (33.33%)		
No	37 (92.50%)	12 (66.67%)		
Whether FN occurs				0.026
Yes	0	3 (16.67%)		
No	40 (100.00%)	15 (83.33%)		
Whether chemotherapy is delayed			*χ*^2^ = 12.35	< 0.001
Yes	5 (12.50%)	11 (61.11%)		
No	35 (87.50%)	7 (38.89%)		
Prolonged time of chemotherapy ^b^			Z = 1.94	0.052
n	5	11		
Median (Q1,Q3)	4.0 (3.0,6.0)	9.0 (7.0,11.0)		
Time to complete radiotherapy ^c^			t = 0.89	0.375
n	40	18		
Mean ± SD	43.55 ± 6.91	45.22 ± 5.80		
Whether bone pain occurs			*χ*^2^ = 1.99	0.159
Yes	1 (2.50%)	3 (16.67%)		
No	39 (97.50%)	15 (83.33%)		

^a^Calculate the duration of 3/4 degree arrhythmia for subjects who have developed 3/4 degree arrhea

^b^Calculate the delay time of chemotherapy for subjects who have delayed chemotherapy

^c^Calculate the duration between the begining and the ending of radiotherapy in the concurrent chemoradiotherapy cycle of the subjects

#### The incidence of grade 4 neutropenia and the incidence of FN

The incidence of grade 4 neutropenia in the experimental and control groups was 7.5 and 33.33%, respectively. The incidence of grade 4 neutropenia in the experimental group was reduced by 25.83%, which was significantly lower compared with the control group (*P* = 0.034). Similarly, FN didn’t occurre in the experimental group (0%) and occurred in 3 patients (16.67%) in the control group, and the difference was also significantly lower in experimental group compared with control group (*P* = 0.026) (Table 3).

#### Time to complete radiotherapy, delay rate of chemotherapy and prolonged time of chemotherapy

There was no statistically significant difference in the mean ± SD time to complete radiotherapy (*P* = 0.375), which was 43.55 ± 6.91 days in the experimental group and 45.22 ± 5.80 days in the control group. Besides, the delay rate of chemotherapy in the experimental group (12.5%) was significantly lower than that of the control group 61.11% (*P* < 0.001). The average prolonged time of chemotherapy in the experimental group was 4.8 days, and the control group was 8.9 days. Although the experimental group had a decreasing trend compared with the control group, the difference was not statistically significant (*P* = 0.052) (Table 3).

### Safety

#### Adverse events

The total incidence of adverse reaction events in the experimental group and the control group were 22/40 (55%) and 17/18 (94.44%), respectively, indicating PEG-rhG-CSF reduced side effects (*P* = 0.003). Of adverse reactions across the two groups, the higher reported adverse events included vomiting, bone marrow suppression, diarrhea, and fever (Table 4).

**Table 4:** Analysis of adverse reactions

Adverse events	Experimental group (*N* = 40)	Control group (*N* = 18)	合计(*N* = 58)
At least one adverse reaction occurred	22 (55.00%)	17 (94.44%)	39 (67.24%)
*χ*^2^	8.77		
*P*	0.003		
Vomiting	17 (42.50%)	10 (55.56%)	27 (46.55%)
Bone marrow suppression	8 (20.00%)	12 (66.67%)	20 (34.48%)
diarrhea	5 (12.50%)	0 (0.00%)	5 (8.62%)
Fever	1 (2.50%)	2 (11.11%)	3 (5.17%)
Anemia	1 (2.50%)	1 (5.56%)	2 (3.45%)
Hypokalemia	1 (2.50%)	0 (0.00%)	1 (1.72%)
Myocardial ischemia	0 (0.00%)	1 (5.56%)	1 (1.72%)
dizziness	1 (2.50%)	0 (0.00%)	1 (1.72%)
Decreased platelets	1 (2.50%)	0 (0.00%)	1 (1.72%)

## Discussion

Concurrent chemoradiotherapy is an important treatment for cervical cancer, but chemoradiotherapy has direct or indirect killing effect on neutrophils, thereby increasing hematological toxicity. RhG-CSF is an effective drug to prevent neutropenia caused by tumor radiotherapy [16]. PEG-rhG-CSF is a PEGylated form of rhG-CSF, which has shown good efficacy and safety in preclinical studies, and it is a long-acting preparation [17, 18]. Studies have shown that PEG-rhG-CSF and rhG-CSF are equally effective in preventing chemotherapy-induced neutropenia [19]. However, there are few studies on the preventive use of PEG-rhG-CSF in concurrent chemoradiation. On this basis, a randomized controlled trial was used to evaluate the efficacy and safety of PEG-rhG-CSF in preventing neutropenia during radiotherapy and chemotherapy of cervical cancer in this study.

The main factor affecting the risk of infection after chemoradiotherapy is the incidence and duration of grade 3–4 neutropenia [20]. In this study, the incidence of grade 3–4 neutropenia in the experimental group was less than that in the control group, and the difference was statistically significant (*P* < 0.05). However, there was no significant difference in the duration of grade 3–4 neutropenia between the test group and the control (*P* = 0.871). In addition, no one in the test group developed FN, and only 3 people in the control group developed FN. The two groups had statistical differences in the incidence of FN (*P* = 0.026). This result is consistent with other research results at home and abroad, indicating that PEG-rhG-CSF has the effect of reducing FN [21, 22].

Besides, compared with the control group, preventive use of PEG-rhG-CSF can also significantly reduce grade 4 neutropenia and the incidence of chemotherapy delay, which is consistent with previous published study [9]. There was no statistical difference in the prolonged time of chemotherapy and the time to complete radiotherapy between the two groups, but the prolonged time of chemotherapy and radiotherapy was statistically shorter in the experimental group compared with that of the control group. This may be related to the use of rhG-CSF for rescue treatment after the occurrence of degree 3 neutropenia in the control group [20] and the small sample size of the research data.

The incidence of adverse reactions between the experimental group and the control group is significantly different in this study. The incidence of adverse reactions in the experimental group is less than that in the control group. The most common adverse reactions of PEG-rhG-CSF in the experimental group were vomiting, bone marrow suppression, diarrhea, and fever [9]. The adverse effects of rhG-CSF are similar to those of conventional doses of rhG-CSF, which reflect the drug characteristics of granulocyte colony stimulating factor [19]. The incidence of vomiting was 42.50%, and the incidence of other adverse reactions was less than 20%, and most of them were less than 5%. This indicates that the adverse events are from the standard treatment (radiotherapy/chemotherapy/chemoradiotherapy) and that this is not due to PEG-rhG-CSF, and that these adverse events are reduced by PEG-rhG-CSF. In addition, due to the long-acting effect of PEG-rhG-CSF, it can reduce the number of subcutaneous injections in the entire process of radiotherapy and chemotherapy, thereby reducing the pain caused by multiple injections [23, 24].

In conclusion, our research showed that PEG-rhG-CSF can be used in concurrent chemoradiotherapy-induced neutropenia preventive therapy. PEG-rhG-CSF has good safety, low incidence of adverse reactions and simple application. A single dose of PEG-rhG-CSF can effectively reduce the occurrence of grade III/IV neutropenia, FN and delayed chemotherapy in patients with cervical cancer chemotherapy. It provides a new option for cervical cancer patients to control neutropenia during chemotherapy.

## Data Availability

The data are available from the corresponding author upon reasonable request.

## References

[1] Arbyn M, Castellsagué X, de Sanjosé S, Bruni L, Saraiya M, Bray F, Ferlay J (2011). Worldwide burden of cervical cancer in 2008. Ann Oncol.

[2] Tilahun Y, Lew C, Belayihun B, Lulu Hagos K, Asnake M (2017). Improving contraceptive access, use, and method mix by task sharing Implanon insertion to frontline health workers: the experience of the integrated family health program in Ethiopia. Glob Health Sci Pract.

[3] Mądry R, Popławska L, Haslbauer F, Šafanda M, Ghizdavescu D, Benkovicova J, Csőszi T, Mihaylov G, Niepel D, Jaeger C, Frkanova I, Macovei A, Staudigl C (2016). Results of a prospective dose intensity and neutropenia prophylaxis evaluation programme (DIEPP) in cancer patients at risk of febrile neutropenia due to myelosuppressive chemotherapy. Wien Klin Wochenschr.

[4] Reducing uncertainties about the effects of chemoradiotherapy for cervical cancer: a systematic review and meta-analysis of individual patient data from 18 randomized trials. J Clin Oncol. 2008;26(35):5802–12. 10.1200/JCO.2008.16.4368 PMC2645100 19001332

[5] Kuderer NM, Dale DC, Crawford J, Lyman GH (2007). Impact of primary prophylaxis with granulocyte colony-stimulating factor on febrile neutropenia and mortality in adult cancer patients receiving chemotherapy: a systematic review. J Clin Oncol.

[6] Lyman GH, Dale DC, Culakova E, Poniewierski MS, Wolff DA, Kuderer NM, Huang M, Crawford J (2013). The impact of the granulocyte colony-stimulating factor on chemotherapy dose intensity and cancer survival: a systematic review and meta-analysis of randomized controlled trials. Ann Oncol.

[7] Giebel S, Thomas X, Hallbook H, Geissler K, Boiron JM, Huguet F, Koller E, Jaeger U, Smedmyr B, Hellmann A, Holowiecki J (2012). The prophylactic use of granulocyte-colony stimulating factor during remission induction is associated with increased leukaemia-free survival of adults with acute lymphoblastic leukaemia: a joint analysis of five randomised trials on behalf of the EWALL. Eur J Cancer.

[8] Kuwabara T, Kobayashi S, Sugiyama Y (1996). Pharmacokinetics and pharmacodynamics of a recombinant human granulocyte colony-stimulating factor. Drug Metab Rev.

[9] CJ XJ, Wang JF, Zhang BH, Zeng XH, Zheng H, Zhang Y, Cai L, Wu YD, Yao Q, Zhao XC, Mao WD, Jiang AM, Chen SS, Yang SE, Wang SS, Wang JH, Pan YY, Ren BY, Chen YJ, Ouyang LZ, Lei KJ, Gao JH, Huang WH, Huang Z, Shou T, He YL, Cheng J, Sun Y, Li WM, Cui SD, Wang X, Rao ZG, Ma H, Liu W, Wu XY, Shen WX, Cao FL, Xiao ZM, Wu B, Tian SY, Meng D, Shen P, Wang BY, Wang Z, Zhang J, Wang L, Hu XC (2018). Advantages with prophylactic PEG-rhG-CSF versus rhG-CSF in breast cancer patients receiving multiple cycles of myelosuppressive chemotherapy: an open-label, randomized, multicenter phase III study. Breast Cancer Res Treat.

[10] Takashi Yoshida SN, Ohtake S, Okafuji K, Kobayashi K, Kondo K, Kanno M, Matano S, Matsuda T, Kanai M, Sugimoto R, Ogawa M, Takaku F (1990). Effect of granulocyte colony-stimulating factor on neutropenia due to chemotherapy for non-Hodgkin's lymphoma. Cancer.

[11] Veronese FM, Pasut G (2005). PEGylation, successful approach to drug delivery. Drug Discov Today.

[12] Lee KHKJ, Lee MH, Han HS, Lim JH, Park KU, Park IH, Cho EK, Yoon SY, Kim JH, Choi IS, Park JH, Choi YJ, Kim HJ, Jung KH, Kim SY, Oh DY, Im SA (2016). A randomized, multicenter, phase II/III study to determine the optimal dose and to evaluate the efficacy and safety of pegteograstim (GCPGC) on chemotherapy-induced neutropenia compared to pegfilgrastim in breast cancer patients: KCSG PC10–09. Support Care Cancer.

[13] Tan H, Tomic K, Hurley D, Daniel G, Barron R, Malin J (2011). Comparative effectiveness of colony-stimulating factors for febrile neutropenia: a retrospective study. Curr Med Res Opin.

[14] Gabrilove JL (2006). An analysis of current neutropenia therapies, including pegfilgrastim. Clin Cornerstone.

[15] Zhai Y, Zhao Y, Lei J, Su Z, Ma G (2009). Enhanced circulation half-life of site-specific PEGylated rhG-CSF: optimization of PEG molecular weight. J Biotechnol.

[16] Zhou CHY, Wang D, An C, Zhou F, Li Y, Chen G, Wu C, He J, Wu G, Song X, Gao J, Liu W, Li B, Shi J, Huang C, Yu J, Feng J, Yue H, Shi M, Xia J (2016). A randomized multicenter phase III study of single Administration of Mecapegfilgrastim (HHPG-19K), a Pegfilgrastim biosimilar, for prophylaxis of chemotherapy-induced neutropenia in patients with advanced non-small-cell lung Cancer (NSCLC). Clin Lung Cancer.

[17] Green MD, Koelbl H, Baselga J, Galid A, Guillem V, Gascon P, Siena S, Lalisang RI, Samonigg H, Clemens MR, Zani V, Liang BC, Renwick J, Piccart MJ, International Pegfilgrastim 749 Study Group (2003). A randomized double-blind multicenter phase III study of fixed-dose single-administration pegfilgrastim versus daily filgrastim in patients receiving myelosuppressive chemotherapy. Ann Oncol.

[18] Vose JM, Crump M, Lazarus H, Emmanouilides C, Schenkein D, Moore J, Frankel S, Flinn I, Lovelace W, Hackett J, Liang BC (2003). Randomized, multicenter, open-label study of pegfilgrastim compared with daily filgrastim after chemotherapy for lymphoma. J Clin Oncol.

[19] Huang W, Liu J, Zeng Y, Wu F, Li N, Chen K, Hong Y, Wang L, Zhen H, Lin L (2018). Randomized controlled clinical trial of polyethylene glycol recombinant human granulocyte colony-stimulating factor in the treatment of neutropenia after chemotherapy for breast cancer. Cancer Chemother Pharmacol.

[20] Sekine ISM, Ito Y, Horinouchi H, Nokihara H, Yamamoto N, Kunitoh H, Ohe Y, Kubota K, Tamura T (2012). Phase I study of concurrent high-dose three-dimensional conformal radiotherapy with chemotherapy using cisplatin and vinorelbine for unresectable stage III non-small-cell lung cancer. Int J Radiat Oncol Biol Phys.

[21] Kourlaba G, Dimopoulos MA, Pectasides D, Skarlos DV, Gogas H, Pentheroudakis G, Koutras A, Fountzilas G, Maniadakis N (2015). Comparison of filgrastim and pegfilgrastim to prevent neutropenia and maintain dose intensity of adjuvant chemotherapy in patients with breast cancer. Support Care Cancer.

[22] Liu F, Du Y, Cai B, Yan M, Yang W, Wang Q (2017). A clinical study of polyethylene glycol recombinant human granulocyte colony-stimulating factor prevention neutropenia syndrome in patients with esophageal carcinoma and lung cancer after concurrent chemoradiotherapy. J Cancer Res Ther.

[23] Beesley VL, Smith DD, Nagle CM, Friedlander M, Grant P, DeFazio A, Webb PM (2018). Coping strategies, trajectories, and their associations with patient-reported outcomes among women with ovarian cancer. Support Care Cancer.

[24] Chunyun Z, Jie S, Wu Y, Shen W, Yongming C, Duanyuan S, Zongpeng z (2017). Pharmacokinetics and pharmacodynamics study of PEGylated recombinant human granulocyte colony-stimulating factor injection in healthy volunteers. Drugs Clin.

